# Is the Pulmonary Embolism Severity Index Being Routinely Used in Clinical Practice?

**DOI:** 10.1155/2015/175357

**Published:** 2015-07-29

**Authors:** Ali Shafiq, Hamza Lodhi, Zaheer Ahmed, Ata Bajwa

**Affiliations:** ^1^Saint Luke's Mid America Heart Institute, 4401 Wornall Road, Kansas City, MO 64111, USA; ^2^University of Missouri-Kansas City, 2411 Holmes Street, Kansas City, MO 64108, USA; ^3^Baptist Desoto Hospital, 7601 Southcrest Parkway, Southaven, MS 38671, USA

## Abstract

*Background*. The Pulmonary Embolism Severity Index (PESI) score can risk-stratify patients with PE but its widespread use is uncertain. With the PESI, we compared length of hospital stay between low, moderate, and high risk PE patients and determined the number of low risk PE patients who were discharged early. *Methods*. PE patients admitted to St. Joseph Mercy Oakland Hospital from January 2005 to August 2010 were screened. PESI score stratified acute PE patients into low (<85), moderate (86–105), and high (>105) risk categories and their length of hospital stay was compared. Patients with low risk PE discharged early (≤3 days) were calculated. *Results*. Among 315 PE patients, 51.7% were at low risk. No significant difference in hospital stay between low (7.11 ± 3 d) and moderate (6.88 ± 2.9 d) risk, *p* > 0.05, as well as low and high risk (7.28 ± 3.0 d), *p* > 0.05, was found. 9% of low risk patients were discharged ≤ 3 days. *Conclusions*. There was no significant difference in length of hospital stay between low and high risk groups and only a small number of low risk patients were discharged from the hospital early suggesting that risk tools like PESI may not have a widespread use.

## 1. Introduction

Pulmonary embolism (PE) is a common contributor to inpatient disease burden in the US with an annual incidence of 69 per 100,000 patients [1]. Although predominantly being treated in an inpatient setting, early discharge and/or outpatient treatment of low risk patient with an acute PE has been shown to be feasible and safe in numerous studies [2–5].

Clinical findings at the time of diagnosis of acute PE can help in prognostic assessment of patients [6, 7] and thus serve as an aid for clinicians to consider outpatient treatment or an early discharge from the hospital. Among the various available tools for prognostication, the “Pulmonary Embolism Severity Index (PESI)” is one of the most extensively validated clinical scores [7–10]. It boasts a high negative predictive value (NPV) of the lowest PESI classes I and II [11, 12] making it a reliable resource to identify low risk PE patients. This can help avoid an unnecessary prolonged hospital stay in a patient who could be a potential candidate for early discharge or closely monitored outpatient anticoagulation therapy.

It is unclear whether clinicians are frequently utilizing risk prediction models such as PESI in a community hospital setting. We wanted to find if there was a difference in the length of hospital stay in patients at low, moderate, and high risks as classified by the PESI score. Furthermore, we wanted to determine the number of low risk patients with acute PE who were discharged early from the hospital.

## 2. Methods

### 2.1. Study Design and Patient Population

This is a retrospective study of patients with acute PE who were admitted to St. Joseph Mercy Oakland Hospital, Pontiac, Michigan, from January 2005 to August 2010. Patients with PE were identified by computer listings of ICD-9 diagnosis codes (415.11, 415.19). The study population included all the adult patients ≥ 18 years of age. Patients who died during the index admission were excluded from the study.

### 2.2. Using the PESI Score and Additional Variables

The PESI score, a well-validated prognostic tool, was utilized to risk-stratify patients with PE into low, moderate, and high risk groups (Supplemental Table  1 in Supplementary Material available online at http://dx.doi.org/10.1155/2015/175357). From the hospital electronic medical record (EMR) data, we abstracted the 11 variables that make up the PESI score. These variables were age, sex, history of cancer, heart failure, chronic lung disease, altered mental status, respiratory rate, heart rate, systolic blood pressure, oxygen saturation, and temperature. The PESI score categorizes patients with PE into classes I (PESI score < 66), II (PESI score 66 to 85), III (PESI score 86 to 105), IV (PESI score 106 to 125), and V (PESI score > 125). We categorized classes I and II as low risk (PESI score ≤ 85), class III as moderate risk (PESI score 86 to 105), and classes IV and V as high risk (PESI score > 105) groups. We also collected some additional variables: brain natriuretic peptide (BNP), troponin I, and D-dimer levels.

The main outcome variable was length of hospital stay in days. As a dichotomous variable, we further determined if any of the low risk patients left the hospital early (≤3 days).

### 2.3. Statistical Analysis

Baseline differences between patients at low, moderate, or high risk for PE (PESI score) were compared by use of Analysis of Variance (ANOVA) for continuous variables and cross tabulation for categorical variables. ANOVA testing was also used to determine if there was a statistically significant difference between the three PESI risk groups (low, moderate, and high) in regard to their respective length of hospital stay. The data fulfilled the most important assumption for ANOVA testing in that the observations were independent. The data further fulfilled most of the other assumptions; the groups were homogenous in terms of their variances. The distributions of the groups approached normality. There were some outliers in the low and high risk groups but these were taken care of by winsorizing. IBM SPSS version 22 was used to run all the analyses.

## 3. Results

### 3.1. Study Group

After applying the inclusion and exclusion criteria, our study group comprised 315 patients, with females comprising 55% of the sample. The mean age of the study group was 63 ± 17 years and the mean length of hospital stay was 7.3 ± 3 days. Among the 315 study patients, 163 (51%) were considered at low risk, 81 (26%) at moderate risk, and 71 (22.5%) at high risk (Table 1).

### 3.2. PESI Risk Groups

**Figure 1:**
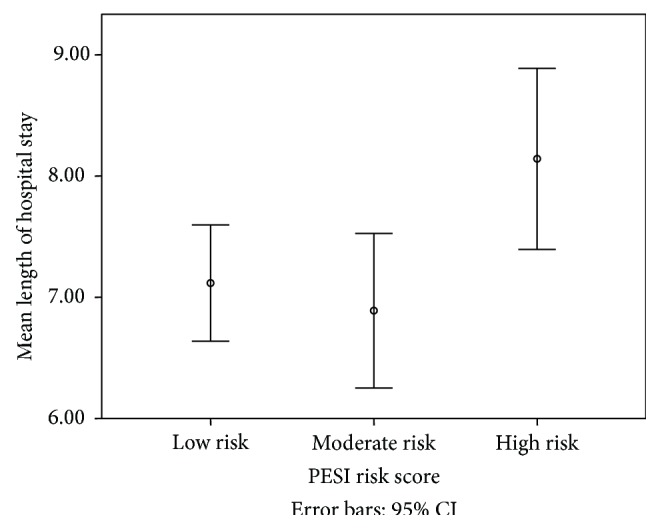
Comparing the length of hospital stay (days) of low, moderate, and high risk PE groups. *X*-axis shows the 3 risk groups: low, moderate, and high, and *Y*-axis shows the mean length of stays with 95% confidence intervals for each of the risk groups. PE: pulmonary embolism; PESI: Pulmonary Embolism Severity Index.

The differences in demographic characteristics between the risk groups can be found in Table 1. The mean length of hospital stay (days ± SD) in the low, moderate, and high risk groups was 7.11 ± 3, 6.88 ± 2.9, and 7.28 ± 3.0, respectively (Table 1, Figure 1). The median length of hospital stay (days) for low, moderate, and high risk groups was 7, 6, and 7, respectively. Low risk PE patients were more likely to be younger (55 y ± 16) and female (60%) and have an early hospital discharge (7.6) but less likely to have a history of heart failure (4%) and cancer (6%) and be hypoxic (0.6%) or hypotensive (1%) as compared to the moderate and high risk PE patients.

### 3.3. Statistical Analysis

By using one-way ANOVA analysis testing, we found that there was a statistically significant difference between the mean lengths of hospital stay in our 3 different pulmonary embolism risk groups stratified by the PESI score with *F*(2,312) = 3.702, *p* = 0.026 (Figure 1). The effect size *d* = 0.01. Furthermore, by using the post hoc test Scheffé to determine further difference between individual groups, we found that there was no statistically significant difference between the mean lengths of stay of the low and moderate risk group (*p* value > 0.05) as well as the low and high risk group (*p* value > 0.05). However, there was a marginal statistically significant difference between the moderate and high risk groups (*p* value = 0.043) in terms of mean length of hospital stay.

### 3.4. Hospital Discharge

**Figure 2:**
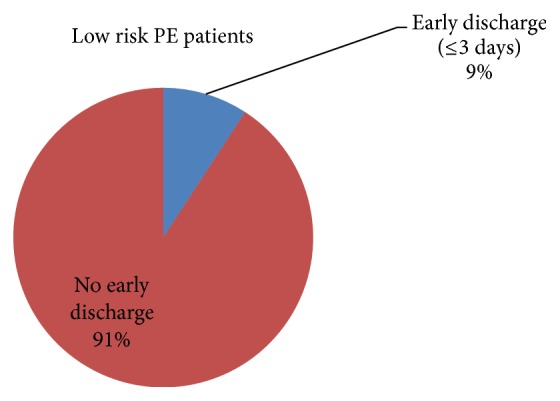
Proportion of low risk PE patients discharged from the hospital early (≤3 days). Pie chart representing all low risk PE patients with a proportion (blue) that shows early discharge (≤3 days). PE: pulmonary embolism.

From among the total study group, 7.6% of patients were discharged early (≤3 days). 15/163 (9%) of the low risk (Figure 2), 7/81 (8%) of the moderate risk, and 2/71 (3%) of the high risk PE patients were discharged from the hospital within 3 days. The reason for prolonged stay beyond 3 days was intravenous (IV) unfractionated heparin (UFH) administration in 134/163 (82%) of the low risk PE patients. In the rest of the low risk patients, 3 stayed for cardiac, 4 for pulmonary, 5 for infectious, and 6 for renal related problems. 11 patients had prolonged stays for miscellaneous reasons.

### 3.5. Presenting Symptoms

The most common presenting symptom in the total study group was dyspnea (43%). Among the risk groups, chest pain was the most common presenting symptom in the low risk PE patients (40%) but dyspnea remained the common presenting symptom in moderate risk (50.6%) and high risk (50.7%) PE patients. The least common presenting symptom in the study group was syncope (6%) but when present it was most commonly seen in high risk (13%) followed by moderate risk (5%) and low risk (3.7%) patients (Table 2).

## 4. Discussion

In this retrospective observational study, we found that the average length of hospital stay for patients who presented with an acute PE was 7 days which was similar to previous reports [13]. By utilizing the PESI score we categorized patients with acute PE into low, moderate, or high risk and compared their length of hospital stay. No significant difference was found between the low risk category and moderate and high risk categories with respect to their length of hospital stay. Furthermore, approximately half of the patients who presented to the hospital with acute PE were at low risk and only 9% of these low risk patients were discharged from the hospital early (within 3 days of their admission).

More and more evidence is emerging where an early discharge from the hospital and/or outpatient treatment of selective low risk PE patients may be safe [14]. Current American College of Chest Physicians (ACCP) guidelines also recommend that low risk patients with PE should be discharged early from the hospital [15]. However, the ideal candidate for early discharge is not well defined with the different risk stratification methods being used. Risk prediction models that have been proposed to identify low risk PE patients are the Hestia criteria, the Geneva score, the Low Risk Pulmonary Embolism Decision rule, the Global Registry of Acute Coronary Events, the PESI score, and the simplified PESI (sPESI) score [16]. Among these risk scores, the PESI and sPESI scores have been opined to be the most reliable in terms of predicting morbidity and mortality in patients who present with an acute PE [16]. The PESI score is based on 11 readily available clinical variables and is a well-validated tool to predict the risk of adverse outcomes at 30 and 90 days after hospital discharge [17]. Patients with PESI classes I and II are categorized as having a low risk of death after discharge [18]. Thus, the PESI score could help us identify the subset of patients with acute PE who may be safely discharged from the hospital early and/or be treated as an outpatient. However, despite the reliable prediction of the PESI score, clinicians may not be making use of this or other risk scores for purposes of arranging early discharge and/or outpatient therapy for low risk patients with PE. In our study there was no significant difference in the length of hospital stay between the low and high risk PE patients. Furthermore, less than 10% of the low risk patients were discharged early.

Our study shows that there is potential for a large patient population that present to the hospital with an acute PE to be discharged early. 50% of patients who presented to our community hospital were found to be at low risk which is similar to previous studies [13]. A vast majority of these low risk PE patients stayed in the hospital to receive IV UFH thus prolonging their hospital stay. However, ACCP guidelines recommend preferring subcutaneous agents such as low-molecular weight heparin (LMWH) or fondaparinux over intravenous UFH (grade 2C for LMWH; grade 2B for fondaparinux) [15]. There are also a number of newer oral anticoagulants such as rivaroxaban and dabigatran that patients can receive for outpatient treatment. Thus, by making use of risk prediction tools such as PESI, clinicians may be able to select a significant number of patients with an acute PE for early discharge and outpatient therapy. Our study has the potential to raise provider awareness of the number of PE patients admitted to a community hospital setting that can be considered for early discharge. This can lead to shorter hospital stay, increase patient satisfaction, and prove to be cost effective.

### 4.1. Limitations

Our study should be interpreted in light of the limitations of a retrospective observational study. First, this was a single center study and we had a fairly small patient population (sample size) making the generalizability of the results across other centers be less certain. Secondly, the diagnosis of acute PE was made through chart review and was not confirmed with results from any diagnostic testing which could have led to misdiagnosis. We could not report information regarding several variables (BNP, D-dimer, and troponin) that we collected because of missing data in the majority of the study population. Furthermore, we did not collect data regarding the type of anticoagulation the patients received, which could have helped provide further information of their length of hospital stay. Finally, we did not look at mortality and other outcome data at follow-up for the risk groups. Future work should include investigating the outcomes of patients with PE who are discharged early as compared to having a prolonged hospital stay.

## 5. Conclusion

In our retrospective study of patients with acute PE, we found no meaningful difference in the length of hospital stay between different risk group categories after risk-stratifying with PESI score. Half of the patients admitted to our community based teaching hospital with acute PE were at low risk but a very small number, that is, 9%, of low risk patients were discharged from the hospital early (within 3 days of admission). This might imply that clinicians are not routinely making use of risk stratification tools such as the PESI score to help guide management and disposition of acute PE patients. Identifying such low risk patient population for early hospital discharge has the potential to decrease hospital stay and be cost effective.

## Supplementary Material

Supplemental Table 1. The upper part of the table shows the predictor variables for the PESI score and their assigned risk scores. The lower part of the table shows classes I to V which represent patients with PE at low to high risk for having adverse events after discharge from the hospital, based on the total PESI score. PESI: Pulmonary Embolism Severity Index; PE: pulmonary embolism.

## Figures and Tables

**Table 1:** Baseline characteristics of total study population as well as risk stratified by PESI.

Variables	Total *n* = 315	Low risk PE *n* = 163	Moderate risk PE *n* = 81	High risk PE *n* = 71
Age (yrs ± SD)	63 ± 17	55 ± 16	72 ± 11	73 ± 14
Male (%)	44	40	43.2	56.3
Medical history				
Heart failure (%)	20.3	4.3	31	45
Cancer (%)	23	6	33	52
CLD (%)	19	10	22	35
Hospital characteristics (%)				
Tachypnea (RR > 30)	4.4	0	1	18
Tachycardia (HR > 100)	13	7.4	11	30
Hypotension (SBP < 100)	7.3	1.2	6.2	22.5
Hypothermia (temp. < 96 F)	2.5	0	1.2	10
Encephalopathy	2	0	0	8.5
Hypoxia (Ox sat < 90%)	6.3	0.6	2.5	24
Early discharge (≤3 days) (%)	7.6	9.2	8.6	3
Hospital stay (days ± SD)	7.3 ± 3	7.1 ± 3	6.8 ± 2.9	8.1 ± 3
Reason for hospital stay (%)				
Anticoagulant bridging	85.6	82	89	90
Other	14.4	18	11	10

CLD: chronic lung disease; HR: heart rate; Ox sat: oxygen saturation; SD: standard deviation.

**Table 2:** Presenting symptoms of the study population and in different PESI risk groups.

Presenting symptom	Total	Low risk	Moderate risk	High risk
Dyspnea (%)	43.50	36.80	50.60	50.70
Chest pain (%)	32.10	40.50	26	20
Leg swelling (%)	7.30	8.60	8.60	3
Syncope (%)	6	3.70	5	13
Other (%)	11.10	10.40	9.90	14
