# Supplementary material for: Is the Pulmonary Embolism Severity Index Being Routinely Used in Clinical Practice?
Source: Thrombosis. 2015 Jul 29;2015:175357. doi: 10.1155/2015/175357 (PMC4532959; doi:10.1155/2015/175357)
Supplement: Supplementary file 1 — Supplemental Table 1. The upper part of the table shows the predictor variables for the PESI score and their assigned risk scores. The lower part of the table shows classes I to V which represent patients with PE at low to high risk for having adverse events after discharge from the hospital, based on the total PESI score. PESI: Pulmonary Embolism Severity Index; PE: pulmonary embolism. [file 175357.f1.pdf]

Supplemental Table 1. PESI SCORE

Predictors	Score
Age	Years
Male	+10
Cancer	+30
CHF	+10
COPD	+10
HR > 110	+20
SBP < 100	+30
RR >/= 30	+20
Body Temp < 36 C	+20
Delirium	+60
Arterial oxygen saturation < 90%	+20

**Total Score**	**Class**
≤ 65	I
66-85	II
86-105	III
106-125	IV
> 125	V

CHF, congestive heart failure; COPD, chronic obstructive pulmonary disease; HR, heart rate; SBP, systolic blood pressure
